# Supplementary material for: A full gap above the Fermi level: the charge density wave of monolayer VS2
Source: Nat Commun. 2021 Nov 25;12:6837. doi: 10.1038/s41467-021-27094-x (PMC8617271; doi:10.1038/s41467-021-27094-x)
Supplement: Supplementary file 1 — Supplementary Information [file 41467_2021_27094_MOESM1_ESM.pdf]

Supplementary Information of “A full gap above the Fermi level: the charge density wave of monolayer VS_2”

Camiel van Efferen[1]*, Jan Berges[2], Joshua Hall[1], Erik van Loon[2], Stefan Kraus[1], Arne Schobert[2], Tobias Wekking[1], Felix Huttmann[1], Eline Plaar[1], Nico Rothenbach[3], Katharina Ollefs[3], Lucas Machado Arruda[4], Nick Brookes[5], Gunnar Schönhoff[2], Kurt Kummer[5], Heiko Wende[3], Tim Wehling[2], Thomas Michely[1]

[1]*II. Physikalisches Institut, Universität zu Köln, Zülpicher Straße 77, 50937 Köln, Germany*
[2]*Institut für Theoretische Physik, Bremen Center for Computational Materials Science, and MAPEX Center for Materials and Processes, Otto-Hahn-Allee 1, Universität Bremen, 28359 Bremen, Germany*
[3]*Fakultät für Physik und Center für Nanointegration Duisburg-Essen (CENIDE), Universität Duisburg-Essen, Carl-Benz-Straße, 47057 Duisburg, Germany*
[4]*Institut für Experimentalphysik, Freie Universität Berlin, Arnimallee 14, 14195 Berlin, Germany*
[5]*European Synchrotron Research Facility (ESRF), Avenue des Martyrs 71, CS 40220, 38043 Grenoble Cedex 9, France*

Supplementary Note 1: Unit cells

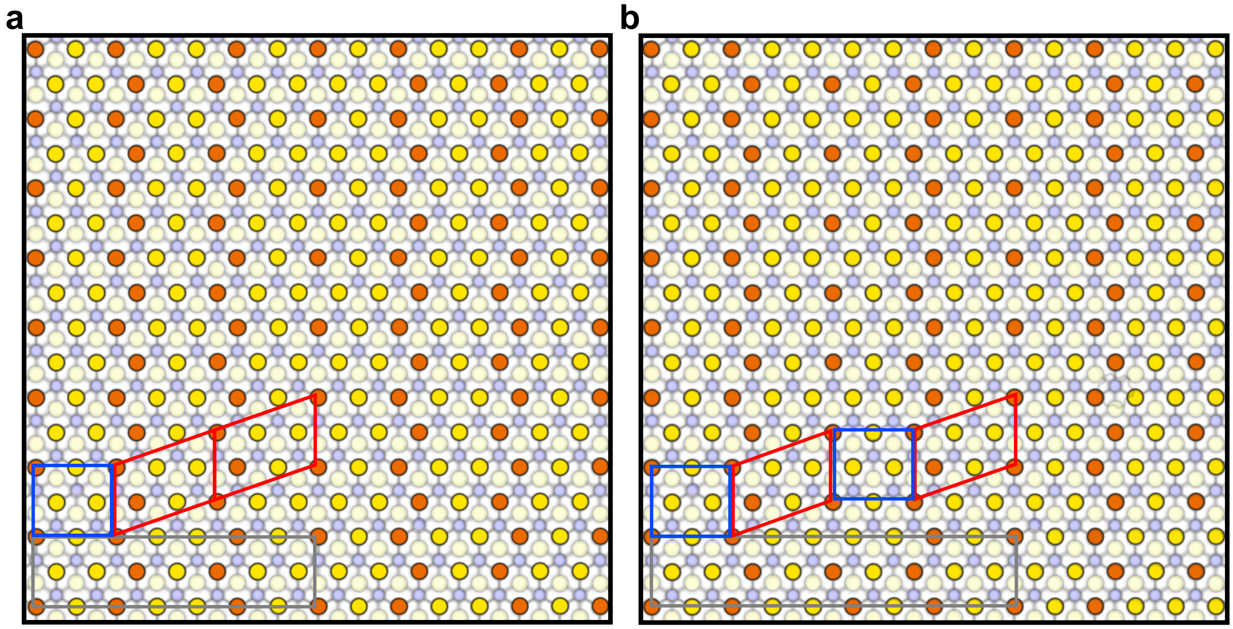

Supplementary Figure 1: Correspondence between unit cells in monolayer VSe_2 literature and the unit cells used in this paper. In **a** and **b**, models of the 1T-VS_2 atomic lattice are depicted with V atoms in blue and bottom-S atoms in faint yellow. $7 \times \sqrt{3}$R30° (**a**) and $9 \times \sqrt{3}$R30° (**b**) superstructures are visible in the top-S atoms, which are drawn in two colors to mimic the experimental apparent height in yellow (bright) and orange (dark). The dark gray rectangles indicate the $7 \times \sqrt{3}$R30° (**a**) and $9 \times \sqrt{3}$R30° (**b**) unit cells. The blue rectangle is a $2 \times \sqrt{3}$R30° unit, the red rhombus a $\sqrt{7}$R19.1° $\times \sqrt{3}$R30°.

In the isotypic material VSe_2, a superstructure of same symmetry as in VS_2, has been identified and attributed to a charge density wave (CDW)[1–4]. In these studies, the superstructure was described by a combination of $2 \times \sqrt{3}$R30° and $\sqrt{7}$R19.1° $\times \sqrt{3}$R30° units, which we mark in our model in Supplementary Figure 1a, b in blue and red, respectively. By the combination of a single $2 \times \sqrt{3}$R30° and two $\sqrt{7}$R19.1° $\times \sqrt{3}$R30° units, the $7 \times \sqrt{3}$R30° CDW lattice can be described; two $2 \times \sqrt{3}$R30° units and two $\sqrt{7}$R19.1° $\times \sqrt{3}$R30° units make up the $9 \times \sqrt{3}$R30° lattice.

Supplementary Note 2: Tip-induced switching between distorted and undistorted phase

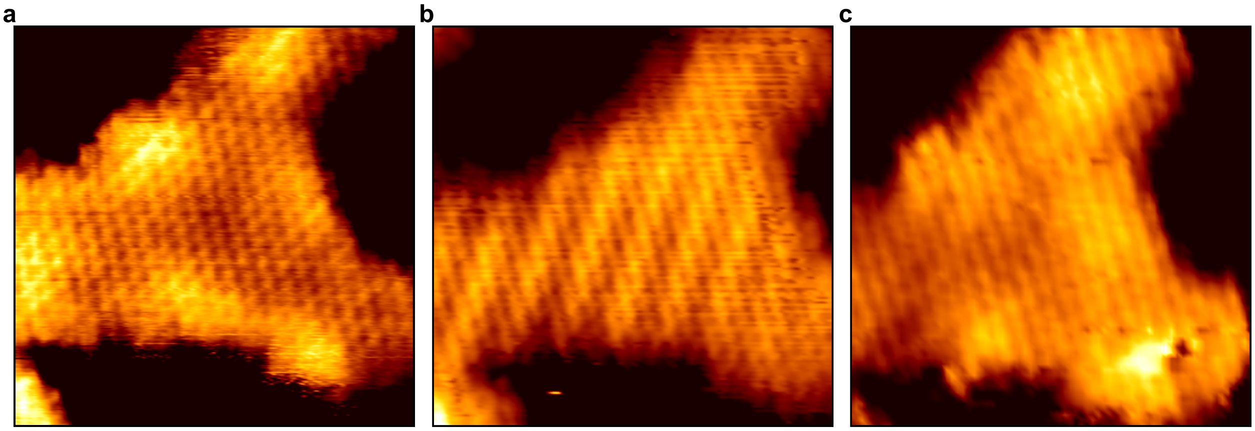

Supplementary Figure 2: Influence of STM tip on monolayer VS_2: the two consecutive STM scans in panel **a** and **b** document the STM-tip-induced switch from the undistorted to the superstructure CDW phase. Scan **c** is taken 15 minutes later and shows no more signs of the superstructure. Images taken at $300\,\mathrm{K}$. Measurement parameters: **a–c** $7 \times 7\,\mathrm{nm}^2$, $I_\mathrm{t} = 0.5\,\mathrm{nA}$, $V_\mathrm{t} = -90\,\mathrm{meV}$.

The presence of the superstructure at room temperature can also be influenced by the scanning tunneling microscope (STM) tip. Supplementary Figure 2a, b shows two consecutive STM scans, taken at the same position, tunnelling current, and bias. In Supplementary Figure 2a, the STM reveals only hexagonal atomic ordering inside the small VS_2 structure. In the successive STM scan in Supplementary Figure 2b, the wave superstructure is observed in the same region, with the phase transition apparently triggered by the interaction with the STM tip. A subsequent STM scan taken about 15 minutes later, displayed in Supplementary Figure 2c, again shows the absence of the superstructure.

Supplementary Note 3: 1T-VSe_2 vs 1T-VS_2 and $9 \times \sqrt{3}R30°$ vs $7 \times \sqrt{3}R30°$

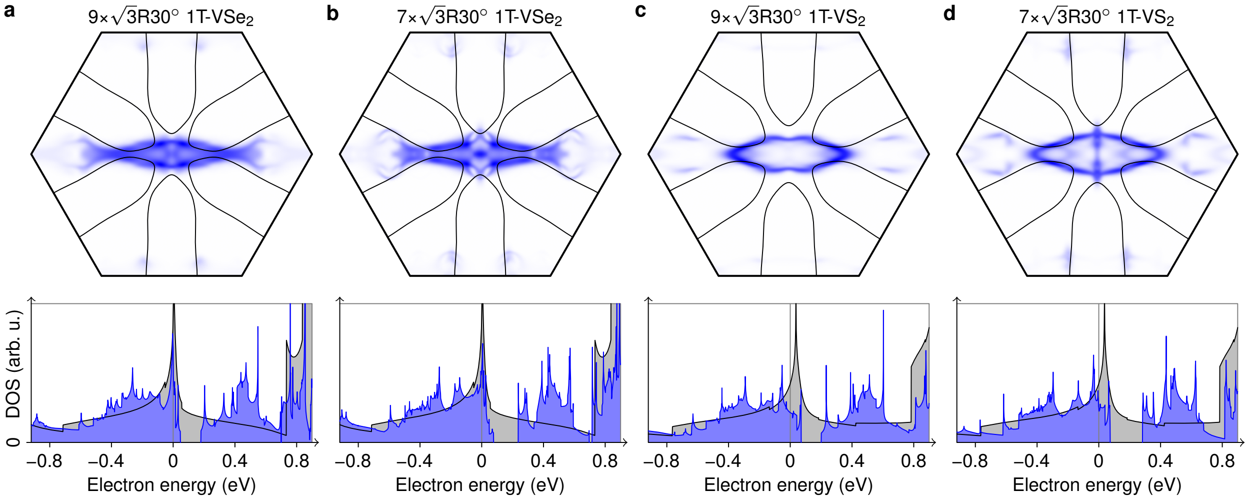

Supplementary Figure 3: Fermi surface and density of states (DOS) of monolayer **a, b** 1T-VSe_2 and **c, d** 1T-VS_2 in the undistorted phase as well as for the **a, c** $9 \times \sqrt{3}R30°$ and **b, d** $7 \times \sqrt{3}R30°$ CDW as obtained from DFT. The CDW data has been unfolded to the Brillouin zone of the undistorted structure. Here, the color saturation corresponds to the overlap of CDW and undistorted wave functions for the same **k** point.

In Supplementary Figure 3a, b, we show the Fermi surface and density of states (DOS) of monolayer 1T-VSe_2 in the undistorted as well as $9 \times \sqrt{3}R30°$ and $7 \times \sqrt{3}R30°$ CDW phases from density functional theory (DFT). In VSe_2 a similar CDW as the one found in VS_2 has been reported repeatedly[1,2,4–6]. As points of reference, corresponding results for monolayer 1T-VS_2 are displayed in Supplementary Figure 3c, d. The results for the $9 \times \sqrt{3}R30°$ and $7 \times \sqrt{3}R30°$ cells agree qualitatively. Furthermore, our calculations suggest that 1T-VSe_2 and 1T-VS_2 are very similar in their electronic structure. In the distorted phase, 1T-VSe_2 will also have a full gap in the unoccupied states; at the Fermi energy, only a partial gap is expected. Though this has been observed in experiment[2,3], most studies on monolayer VSe_2 agree on a full gap located at the Fermi level[1,4,6,7]. To our understanding, such a gap would require a filling of the downwards-dispersing bands near Γ, which are not gapped in the CDW configuration. According to our DFT calculations for both 1T-VS_2 and 1T-VSe_2 on a $9 \times \sqrt{3}R30°$ ($7 \times \sqrt{3}R30°$) supercell, $1/9 \approx 0.11$ ($1/7 \approx 0.14$) additional electrons would shift the gap to the Fermi energy (compare Supplementary Figure 10a, b). This charge could be provided by, e.g., the substrate or defects.

Supplementary Note 4: Nesting conditions and electron–phonon coupling

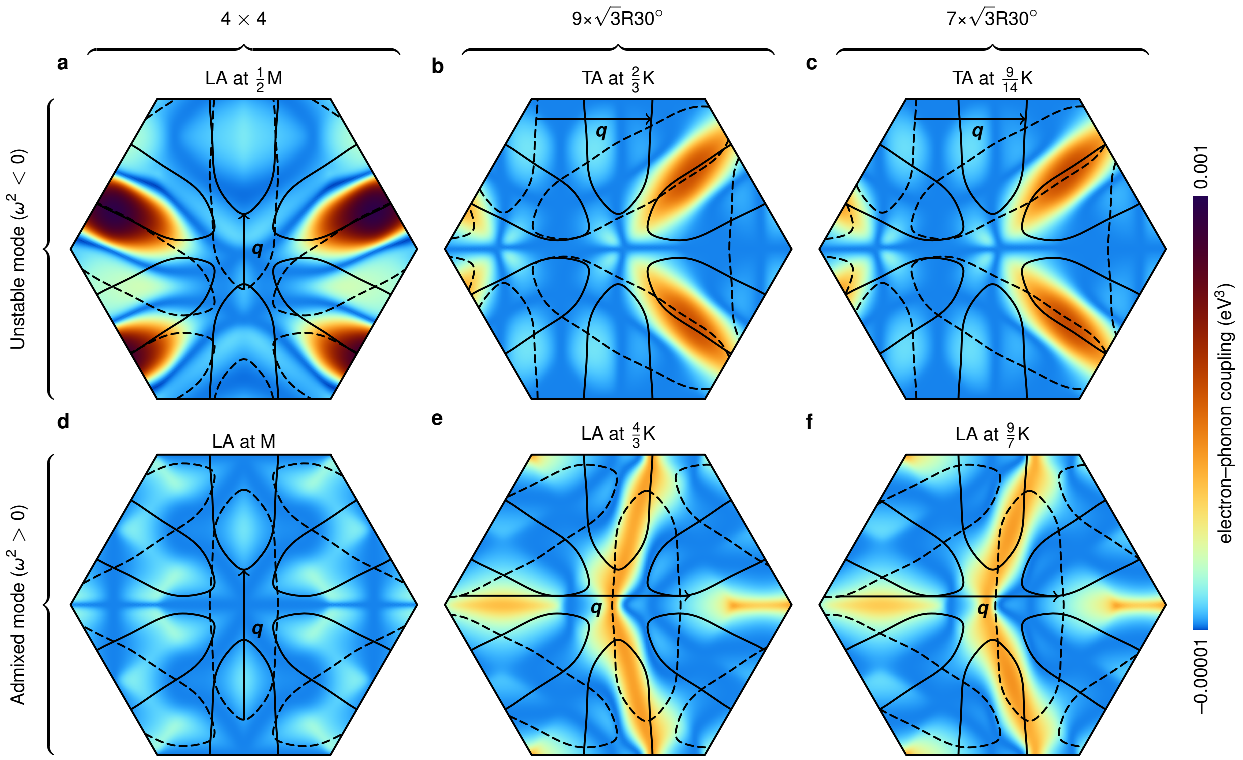

Supplementary Figure 4: Nesting conditions for different longitudinal– and transverse–acoustic (LA and TA) phonon wavevectors $\mathbf{q}$. We show the relevant electron–phonon coupling $2\omega_{\mathbf{q}} g_{\mathbf{k}+\mathbf{q},\mathbf{k}} \widetilde{g}_{\mathbf{k},\mathbf{k}+\mathbf{q}}$ as a function of the electron wavevector $\mathbf{k}$ (color scale) together with the original Fermi surface (solid lines) and the Fermi surface shifted by $-\mathbf{q}$ (dashed lines). Nesting parts of the Fermi surface can only have a strong effect on the phonons if they occur in $\mathbf{k}$-space regions with significant electron–phonon coupling (dark/brown spots). While the $\widetilde{g}$ from DFPT is fully screened, the partially screened g from constrained DFPT (cDFPT)[8] excludes low-energy electronic screening (precisely, from within the isolated band at the Fermi level). Together with the bare electronic susceptibility χ_0, they determine the phonon self-energy $\Pi = g^* \chi_0 \widetilde{g}$ responsible for the instabilities in the phonon dispersion. This analysis is equivalent to the fluctuation diagnostics in Ref. 9. The electron–phonon coupling has been obtained via the EPW code[10,11].

In Supplementary Figure 2a of the manuscript, we can observe two main instabilities in the acoustic phonon dispersion of monolayer 1T-$VS_2$from density functional perturbation theory (DFPT): one in the longitudinal branch at $\mathbf{q} \approx 1/2\,\overline{\Gamma\mathrm{M}}$ and one in the transverse branch at $\mathbf{q} \approx 2/3\,\overline{\Gamma\mathrm{K}}$. A mode with momentum $\mathbf{q}$ will be favoured if there is a large electron–phonon coupling matrix element connecting momenta $\mathbf{k}$ and $\mathbf{k} + \mathbf{q}$ close to the Fermi surface. These nesting conditions are investigated in Supplementary Figure 4. In the longitudinal case, shown in Supplementary Figure 4a, we have almost perfect Fermi-surface nesting together with a strong electron–phonon coupling (cf. Fig. 5c, d of Ref. 12 for the case of 1T-VSe_2). This $\mathbf{q}$ point is compatible with the formation

of a 4×4 CDW, as found, e.g., in bulk VSe_2[13]. Interestingly, despite these favorable conditions, this is not the preferred ground state of monolayer VS_2. Instead, a CDW with a wavevector near $\mathbf{q} = 2/3\,\overline{\Gamma\mathrm{K}}$ and $\mathbf{q} = 9/14\,\overline{\Gamma\mathrm{K}}$ develops, which features only approximate nesting and a slightly reduced coupling strength, as seen in Supplementary Figure 4b, c. As discussed in the main text, the formation of the CDW can only be understood considering non-linear mode–mode coupling. Phonon modes that appear stable in the harmonic approximation contribute significantly to the final atomic displacements, especially the longitudinal–acoustic modes for twice the momenta of the unstable modes, i.e., $\mathbf{q} = \mathrm{M}$, $\mathbf{q} = 9/7\,\overline{\Gamma\mathrm{K}}$, and $\mathbf{q} = 4/3\,\overline{\Gamma\mathrm{K}}$, see Supplementary Figure 4d–f. For both the harmonic (Supplementary Figure 4b, c) and the higher-order contributions (Supplementary Figure 4e, f) to the experimentally observed CDW, we find a similar situation of partially overlapping Fermi pockets in $\mathbf{k}$-space regions of considerable coupling, except that different pairs of pockets are involved.

Supplementary Note 5: Born–Oppenheimer energy surface in TaS_2

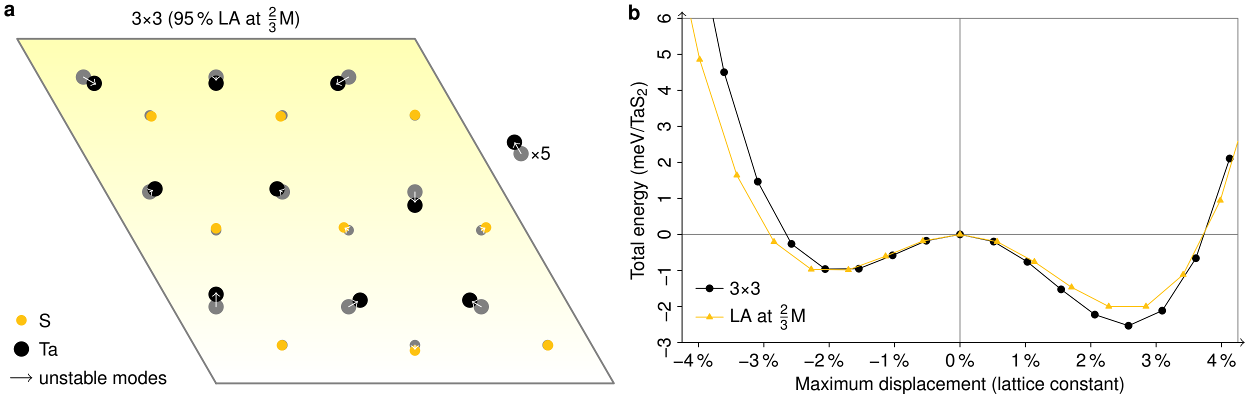

Supplementary Figure 5: a CDW distortion in monolayer 2H-TaS_2 and **b** corresponding Born–Oppenheimer energy surface. Full circles indicate atomic positions and energies for displacements in the direction of the relaxed structure, arrows and triangle marks those for the projection onto the unstable longitudinal–acoustic (LA) phonon modes at the six wavevectors $\mathbf{q} = 2/3\,\overline{\Gamma\mathrm{M}}$. The relaxed atomic displacements have been amplified by a factor of five for better visibility.

While the experimentally observed CDW in VS_2 can only be explained by the nonlinear coupling between soft and stable phonon modes, the 3×3 CDW in the trigonal–prismatic TMDCs is an example of a lattice instability that is determined essentially by a single unstable phonon mode. Analogous to Supplementary Figure 2b–e, Supplementary Figure 5 shows the 3×3 CDW structure and the corresponding Born–Oppenheimer energy surface of monolayer 2H-TaS_2 from DFT: Here, the distortion along the leading unstable phonon normal-mode coordinate largely captures the energy gain associated with the full CDW relaxation. We used the same computational parameters as in Ref. 9.

Supplementary Note 6: Phonon dispersion, nesting conditions, and electron–phonon coupling in 1T-VTe_2

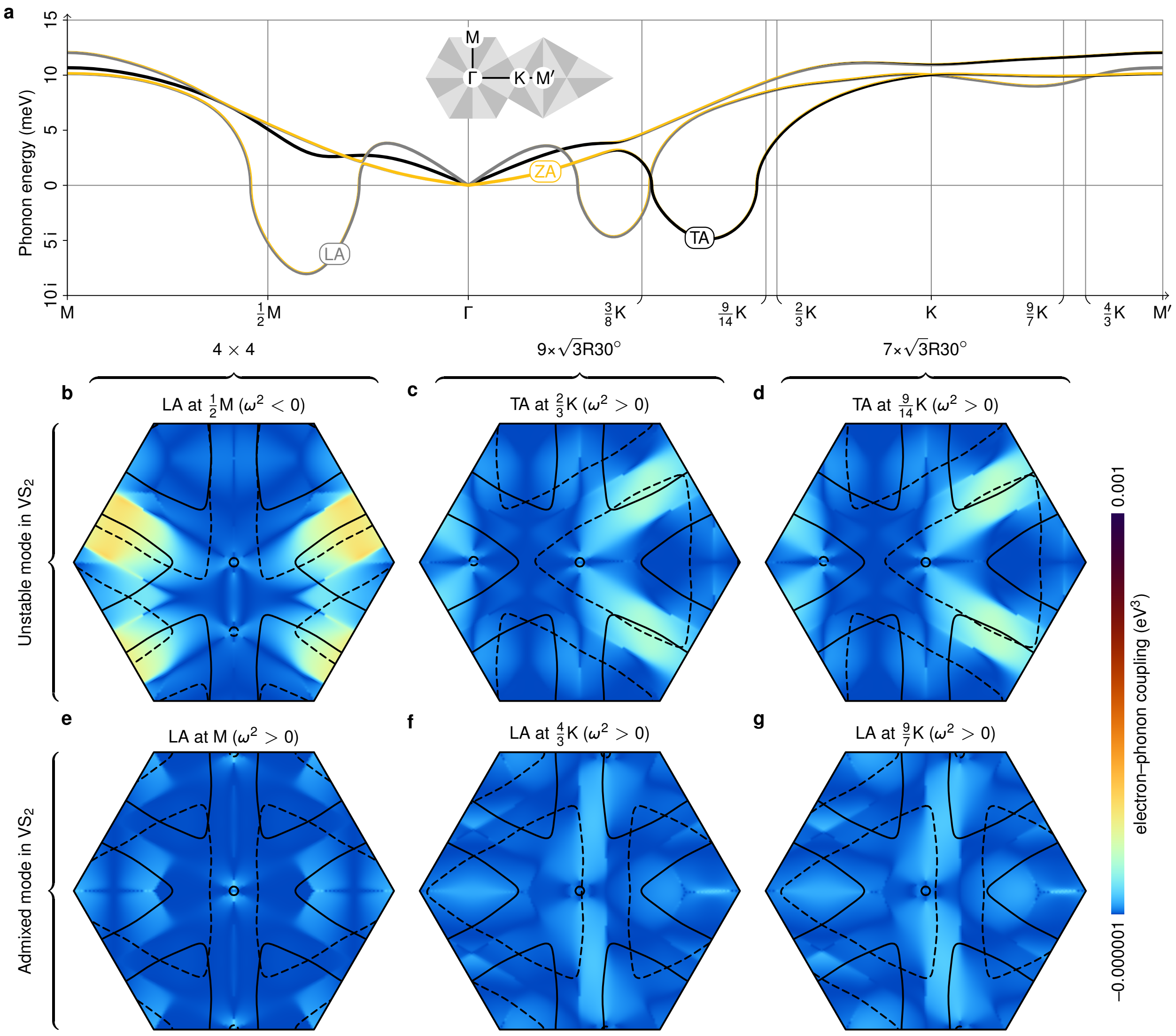

Supplementary Figure 6: a Longitudinal–, transverse– and z–acoustic (LA, TA and ZA) phonon dispersion of monolayer 1T-VTe_2. **b–g** Corresponding electron–phonon coupling $2\omega_{\mathbf{q}} g_{\mathbf{k}+\mathbf{q},\mathbf{k}} \widetilde{g}_{\mathbf{k},\mathbf{k}+\mathbf{q}}$ together with the **k** and **k** + **q** Fermi surfaces (cf. Supplementary Figure 4).

The phonon dispersion of monolayer 1T-VTe_2 obtained from DFPT is shown in Supplementary Figure 6a. We find similar lattice instabilities as in the case of 1T-VS_2 (cf. Supplementary Figure 2a of the main text), albeit shifted to smaller $|\mathbf{q}|$. This shift, which is more pronounced for the transverse–acoustic instability in the $\overline{\Gamma \mathrm{K}}$ direction than for the longitudinal–acoustic instability in the $\overline{\Gamma \mathrm{M}}$ direction, can be traced back to differences in the Fermi surface (topology) rather than in the electron–phonon coupling, see Supplementary Figure 6b–g: Instead of the cigar-shaped electron pockets around the M points in 1T-VS_2 (cf. Supplementary Figure 4), we find triangular

hole pockets around the K points (as well as a small hole pocket at Γ) in 1T-VTe_2. In the latter case, the approximately parallel segments of the Fermi surface are closer together.

Supplementary Note 7: CDW energy gains in VS_2 vs VTe_2

Supplementary Table 1: Comparison of maximum atomic displacements and energy gains upon CDW formation for different materials and periodicities from DFT (PBE). All energies refer to a single $\mathrm{V}X_2$ unit; the reference for the displacements is the lattice constant.

	1T-VS_2		1T-VTe_2	
4×4	$4\,\%$	$3.4\,\mathrm{meV}$	$13\,\%$	$34.2\,\mathrm{meV}$
$7 \times \sqrt{3}\mathrm{R}30^\circ$	$8\,\%$	$22.7\,\mathrm{meV}$	$6\,\%$	$2.5\,\mathrm{meV}$

To compare the energy gains associated with CDW formation in 1T-VS_2 and in 1T-VTe_2 for the different periodicities, we performed structural relaxations on corresponding supercells in the framework of DFT (PBE). The energy gains reported in Table 1 show that in 1T-VS_2 the $7\times\sqrt{3}\mathrm{R}30^\circ$ CDW is favored over the 4×4 CDW; in 1T-VTe_2 vice versa. The DFT prediction is thus in line with experimental observation for both materials.

Supplementary Note 8: Compression of electronic spectrum

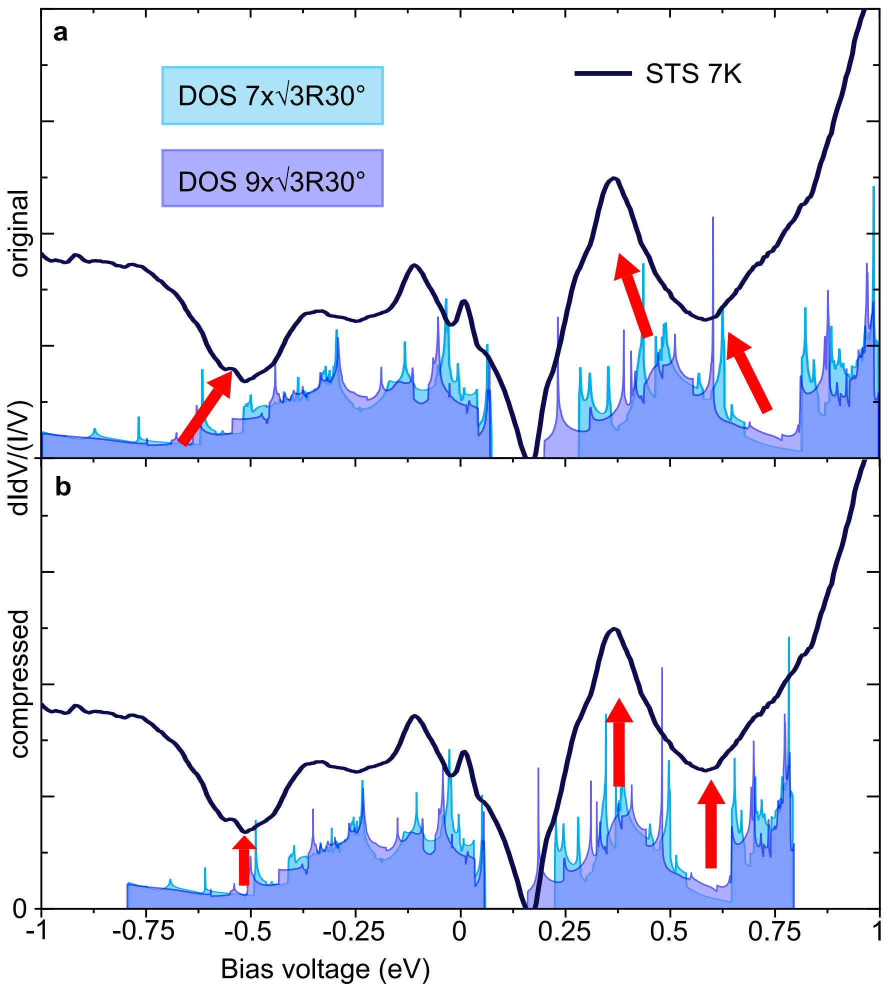

Supplementary Figure 7: Compression of experimental scanning tunneling spectroscopy (STS) data relative to density-functional theory (DFT)-calculated density of states (DOS). In **a**, the $7\,\mathrm{K}$ spectrum from the main manuscript is compared to the calculated DOS for the $7 \times \sqrt{3}\mathrm{R}30°$ and $9 \times \sqrt{3}\mathrm{R}30°$ unit cells. In **b**, the calculated DOS is compressed to about $80\,\%$ of its original width. The red arrows in **a, b** indicate three major features in the spectrum and DOS that can be harmonized between them when the DOS is compressed.

In Supplementary Figure 4a of the main text, the DOS from DFT appears to be wider than the experimental spectrum. Dynamic electronic correlation effects beyond DFT are a possible source of this discrepancy, since they can lead to band renormalization[14]. More precisely, they effect a mass enhancement of the electrons, i.e., the quasi-particle dispersions become flatter than what is expected from theories like DFT. In the case of purely local correlations[15], this effect is described by a single renormalization factor Z or the corresponding mass enhancement factor $1/Z$. Supplementary Figure 7 shows that we obtain a good match between experimental and theoretical spectra by setting $Z = 0.8$. This is indicative of moderate electronic correlations. For comparison, examples range

from diverging mass enhancement at Mott–Hubbard transitions, via mass enhancement factors of about 10 to 1000 in Kondo or heavy fermion systems, to enhancement factors between 1 and 10 in transition-metal compounds like metallic chromium or iron-based superconductors. The mass enhancement factor of $1/Z \approx 1.25$ puts VS_2 at similar electronic correlation strengths as, e.g., metallic chromium[16].

The rise in the normalized $\mathrm{d}I/\mathrm{d}V$ beyond $-0.5\,\mathrm{eV}$ can be attributed to contributions from the graphene/Ir(111) substrate, which can come to dominate the signal for large V when the VS_2 has a small DOS. In this case, the graphene spectrum (not shown) diverges beyond the Ir(111) surface state at $-190\,\mathrm{meV}$.

Supplementary Note 9: Suppression of CDW Fourier intensity within the gap

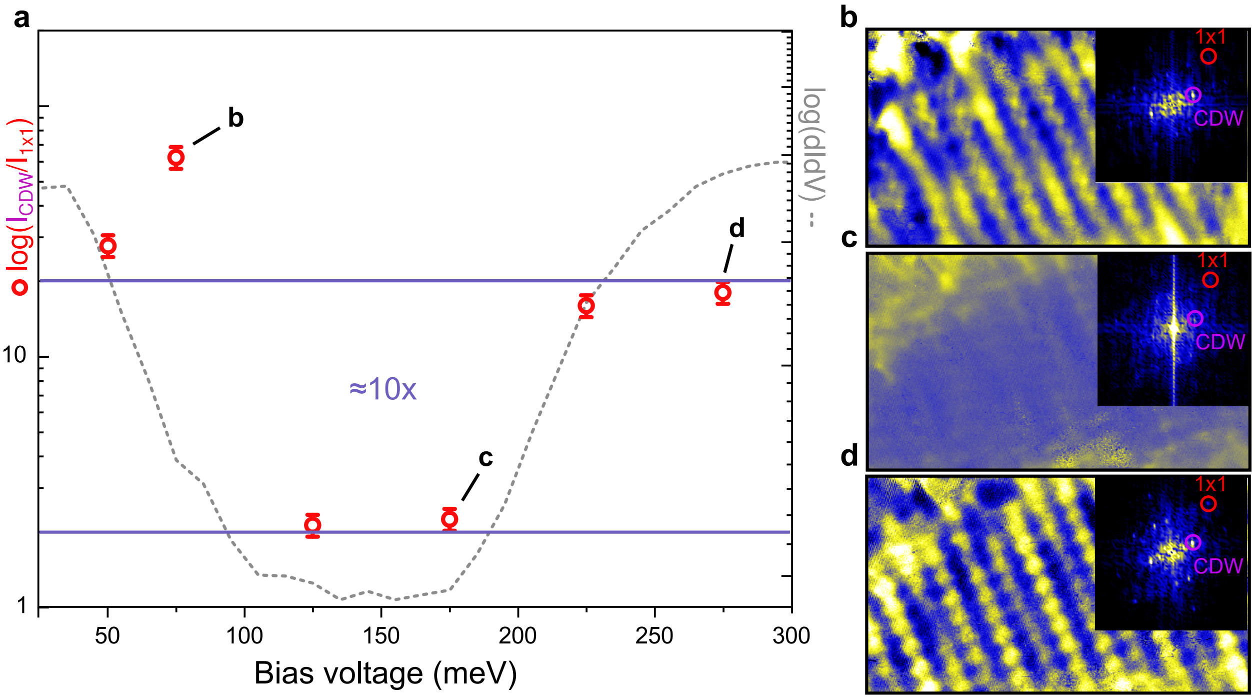

Supplementary Figure 8: Suppression of charge density wave (CDW) within the gap. **a** Logarithmic plot of the CDW intensity in the Fourier transform of $\mathrm{d}I/\mathrm{d}V$ conductance maps, normalized to the 1×1 lattice intensity. Additionally, a $\mathrm{d}I/\mathrm{d}V$ spectrum is plotted in order to indicate the location and width of the gap. **b–d** $\mathrm{d}I/\mathrm{d}V$ conductance maps taken at the voltages indicated in **a**. Measurement settings: (maps) $9.5 \times 5.5\,\mathrm{nm}^2$, $I_\mathrm{t} = 0.3\,\mathrm{nA}$, except for the map at $50\,\mathrm{meV}$, which is taken at $I_\mathrm{t} = 0.6\,\mathrm{nA}$; ($\mathrm{d}I/\mathrm{d}V$ spectrum) $f = 777.7\,\mathrm{Hz}$, $I_\mathrm{t} = 0.4\,\mathrm{nA}$, $V_\mathrm{r.m.s.} = 6\,\mathrm{meV}$. All data taken at $T = 7\,\mathrm{K}$.

Apart from the different charge distributions on either side of the gap discussed in the main manuscript, $\mathrm{d}I/\mathrm{d}V$ maps taken within the gap show a clear suppression of the CDW. For a quantitative analysis, we have Fourier analyzed the $\mathrm{d}I/\mathrm{d}V$ maps and normalized the CDW peak in the Fourier spectrum with respect to the 1×1 lattice peak intensity. The resulting value $R = I_\mathrm{CDW}/I_{1\times1}$ is observed to fall by an order of magnitude within the gap. Since a gap of other than CDW origin would have the same value of R in- and outside of the gap region[17], this is another clear indication of the relation between gap and CDW.

Supplementary Note 10: Bands along extended Brillouin-zone path

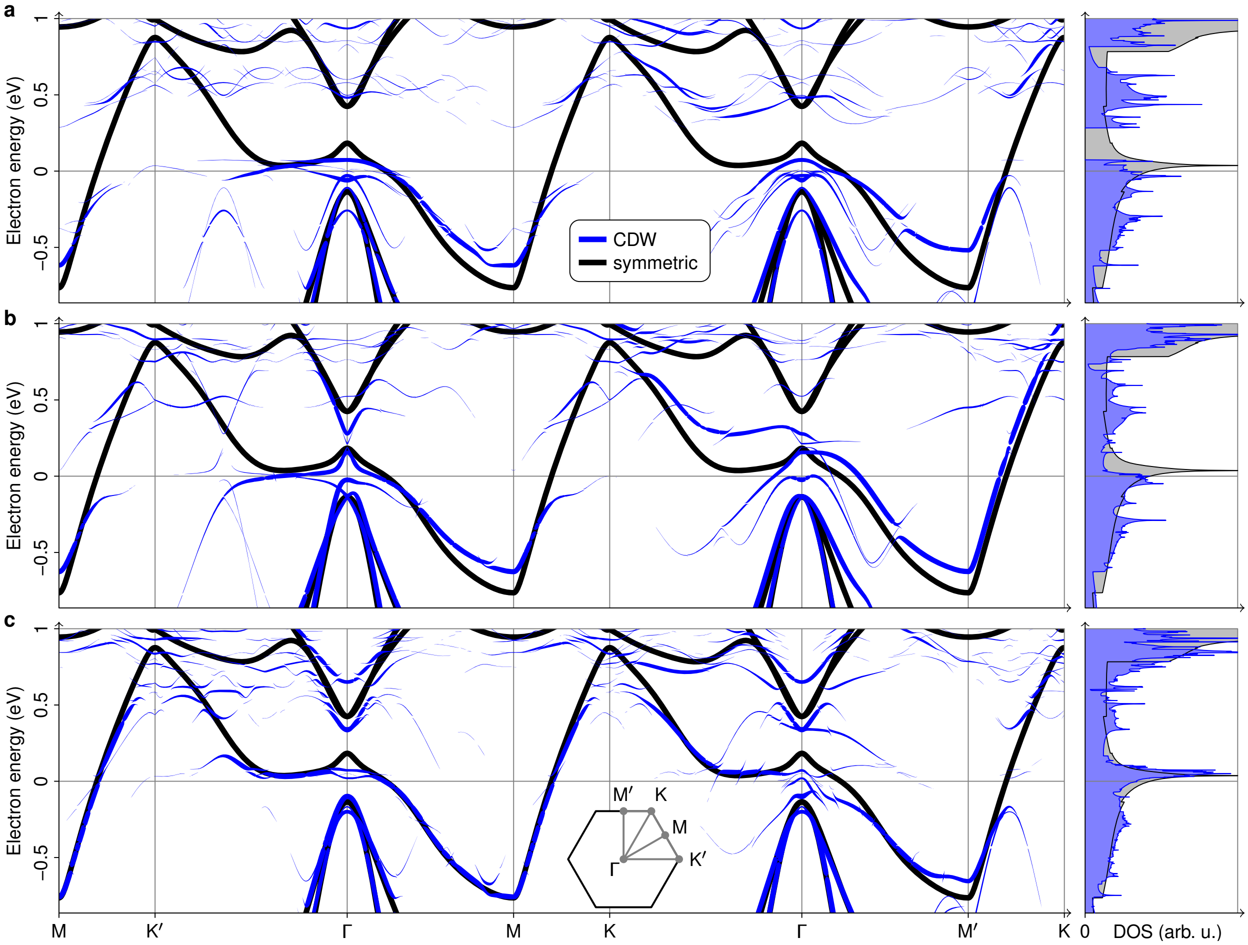

Supplementary Figure 9: Electronic band structure along an extended Brillouin-zone path and density of states (DOS) of monolayer 1T-VS_2 for **a** the full $7 \times \sqrt{3}\mathrm{R}30^\circ$ charge density wave (CDW) displacements, **b** their projection onto unstable phonon modes, and **c** the orthogonal complement.

In Supplementary Figure 5a of the manuscript, we show the electronic band structure of monolayer 1T-VS_2 in the $7 \times \sqrt{3}\mathrm{R}30^\circ$ phase along a selected high-symmetry path Γ–M–K–Γ of the undistorted phase only. Once the distortion breaks the C_3 symmetry, this path is not representative of the full Brillouin zone anymore. For completeness, in Supplementary Figure 9a, we thus reproduce the respective data along an extended path, again supplemented with the DOS. In Supplementary Figure 9b, c, we show the analogous results for the projection of the displacement onto the soft transverse–acoustic phonon modes at $\mathbf{q} = \pm 9/14\,\overline{\Gamma\mathrm{K}}$ and the orthogonal complement. There are some salient differences between the electrons for the full and partial CDW displacement. In Supplementary Figure 9b, the gap between M' and K is missing; in turn, in Supplementary Figure 9c, there is no gap between M and K' as well as M and K. The combination of both displacement components is needed to open a full gap.

Supplementary Note 11: Preservation of states at the Fermi level

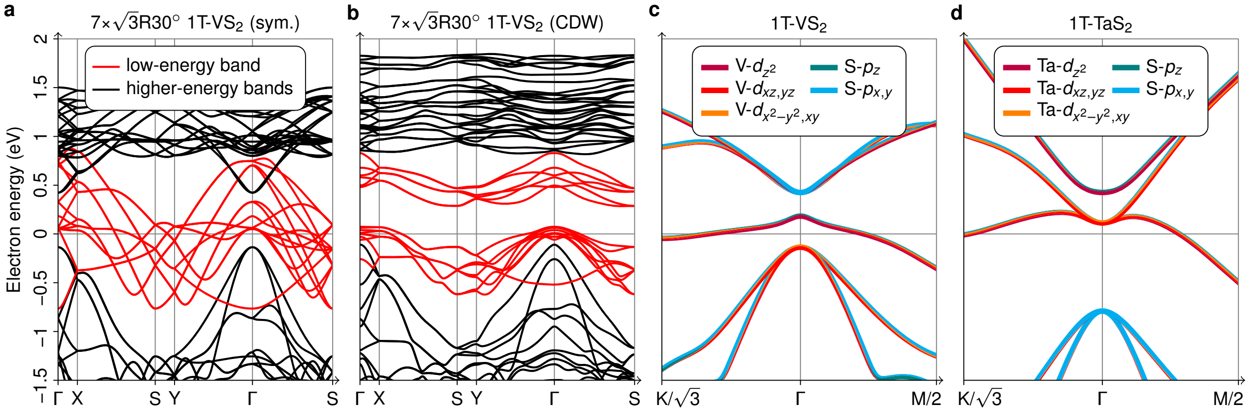

Supplementary Figure 10: a, b Band structure of symmetric and distorted 1T-VS_2 along path through Brillouin zone corresponding to $7 \times \sqrt{3}$R30° cell. The low-energy band is half-filled and splits into seven four-fold (including spin) bands between X and S. Thus, there must be 1/7 unoccupied states per V atom below the gap. **c, d** Orbital-resolved low-energy electron dispersion of 1T-TaS_2 and 1T-VS_2 near Γ. In the case of 1T-VS_2 there is an avoided crossing between V-d and S-$p_{x,y}$ bands.

The CDW does not create a complete gap at the Fermi level. For the commensurate structures used to approximate the incommensurate CDW, it is the combination of the electron count and the symmetry of the unit cell that guarantees a partially filled band, i.e., a metallic DOS. A complete gap at the Fermi level in the CDW would require the splitting of bands that must be degenerate by symmetry of the CDW structures (Supplementary Figure 10a, b), i.e., further symmetry breaking.

The particular form of the remaining spectral weight at the Fermi level resembling a downwards dispersing parabola around Γ in the CDW state (cf. Supplementary Figure 5) can be understood in terms of orbital band characters: In 1T-VS_2, we find an avoided crossing of V-d and S-$p_{x,y}$ bands in the relevant region and thus a signification hybridization between these states (Supplementary Figure 10c). This is opposed to, e.g., the case of 1T-TaS_2, where the S-$p_{x,y}$ states are much lower in energy (Supplementary Figure 10d). Now, while the d-type bands are heavily reconstructed due to the CDW, the p orbitals are less affected and can contribute to a new Fermi surface in the case of 1T-VS_2 in contrast to 1T-TaS_2.

Supplementary Note 12: XMCD sample morphology

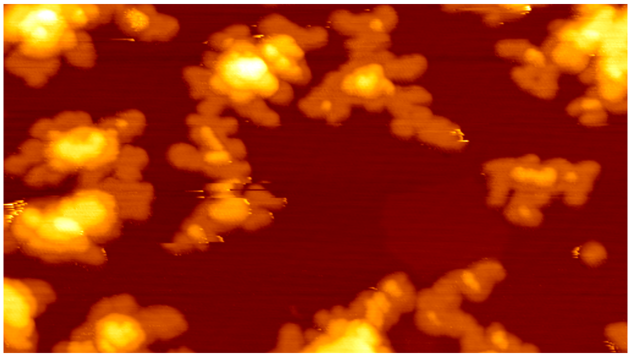

Supplementary Figure 11: Magnetic properties of VS_2: STM topograph illustrating the sample morphology of the XMCD measured sample. Image size: $100 \times 50\,\mathrm{nm}^2$.

In the main manuscript, we describe the magnetic properties of VS_2 as measured by x-ray absorption spectroscopy (XAS) and x-ray magnetic circular dichroism (XMCD). Supplementary Figure 11 displays the sample morphology of the investigated sample. Like the samples shown in the main text, the island shape is dendritic. By comparison to substrate step edges, the monolayer height is measured to be $7\,\text{Å}$. The sample has a monolayer coverage of about $40\,\%$. Distinct height levels indicate up to three layers, with multilayer VS_2 making up approximately $25\,\%$ of the total amount of VS_2 present on the surface.

Supplementary Note 13: DOS for SDW

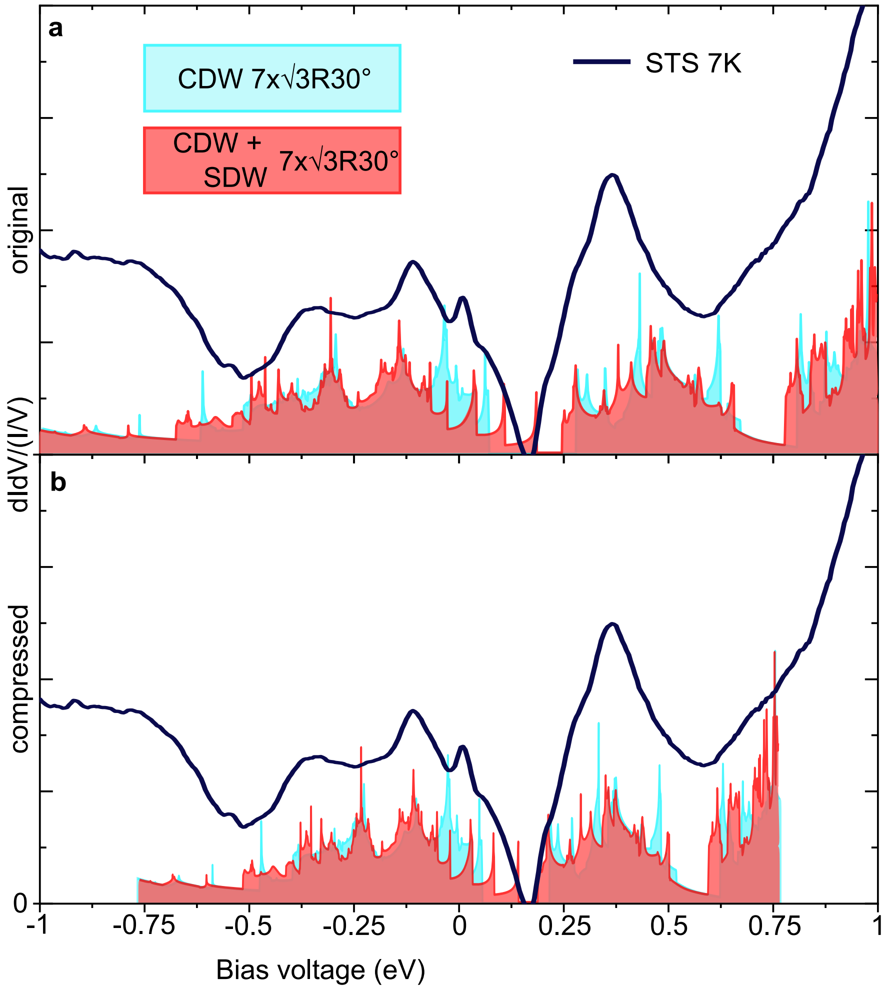

Supplementary Figure 12: **a** Scanning tunneling spectroscopy (STS) data taken with a Au tip on monolayer VS_2 at $7\,\mathrm{K}$ along with density-functional theory (DFT) calculated charge density wave (CDW) density of states (DOS) with (red) and without (cyan) SDW. **b** The same data after compressing calculated DOS to 80% of original size.

In Supplementary Figure 12a, we show the DOS of the CDW structure with and without spin density wave (SDW), along with the experimental $\mathrm{d}I/\mathrm{d}V$ spectra. The most prominent difference in the DOS is the reduction of the gap size. For the $7 \times \sqrt{3}\mathrm{R}30^\circ$ structure, the gap shrinks from $0.21\,\mathrm{eV}$ to $0.06\,\mathrm{eV}$ when the CDW is coupled to a SDW. Since the CDW gap is indeed much larger in DFT than the experimental gap, this can be considered as an additional argument for the simultaneous presence of a SDW. Taking into account the compression of the experimental data, discussed under Supplementary Figure 7, the DOS of the coupled CDW–SDW is in even better agreement with the experimental spectra, as seen in Supplementary Figure 12b.

Supplementary Note 14: Comparison of DFT results with literature data for VSe2

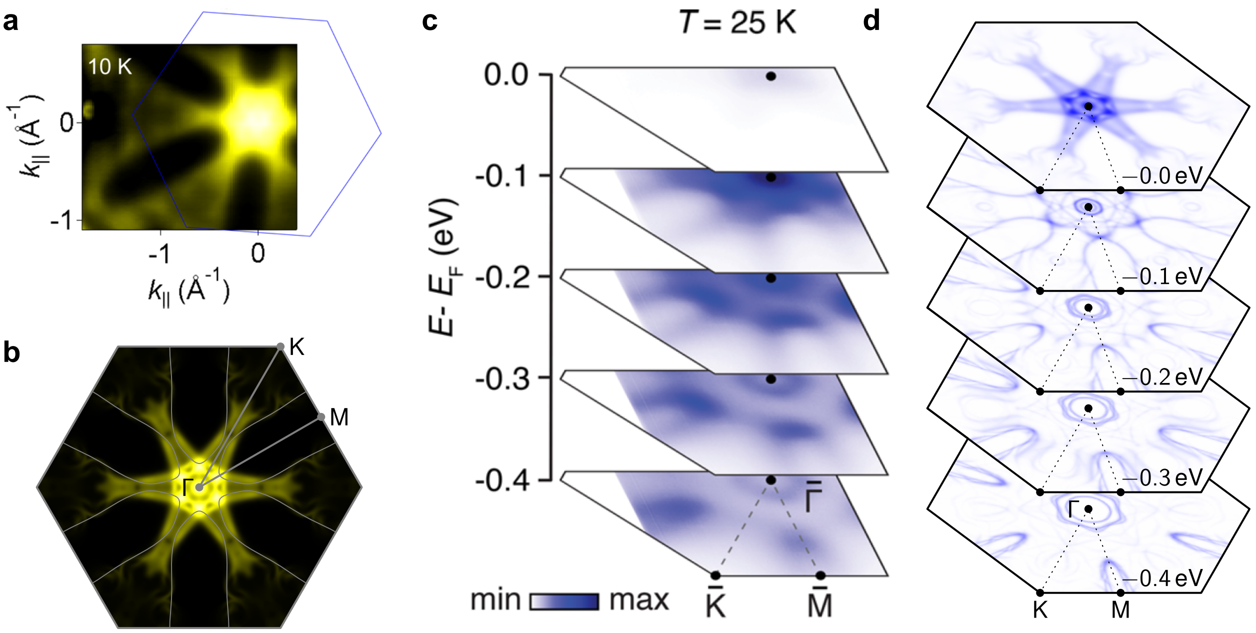

Supplementary Figure 13: Fermi surface of $7 \times \sqrt{3}\mathrm{R}30°$ CDW of monolayer 1T-VSe_2 averaged over regions of different CDW orientations and comparison to experiment. **a** Fermi surface reprinted with permission from Ref. 2 © 2018 American Physical Society. **b** Symmetrized Fermi surface calculated in this work. **c** Energy isolines reprinted with permission from Ref. 7 © 2018 American Chemical Society. **d** Symmetrized energy isolines calculated in this work.

In order to compare our DFT results for monolayer VSe_2 to existing ARPES studies, we have averaged our calculated Fermi surface of VSe_2 in the $7 \times \sqrt{3}\mathrm{R}30°$ CDW phase over all domains of the CDW with respect to the lattice. In this way, many of the familiar ARPES characteristics of VSe_2 are uncovered. If we compare to VSe_2 systems where only the $7 \times \sqrt{3}\mathrm{R}30°$ CDW was observed[2,3], we see that the experimental data in Supplementary Figure 13a, taken from Ref. 2, shows excellent agreement with our calculated Fermi surface in Supplementary Figure 13b. In particular, we observe the formation of gaps between M and K, while the rest of the Fermi surface remains intact. The apparent persistence of the six cigar-shaped electron pockets and the appearance of the hole pocket around Γ are visible in both theory and experiment. That the ARPES measurements show such small changes during the transition to the CDW phase can therefore be understood as stemming mostly from the fact that it is an averaging technique. More subtle changes to the band structure around Γ and the elliptic electron pockets cannot easily be compared by eye. All in all, our theoretical studies match very well to experimental ARPES reports of VSe_2 in the $7 \times \sqrt{3}\mathrm{R}30°$ phase[2,3].

It must however be noted that most publications on VSe_2 find, in contrast to our prediction, a full gap at the Fermi level[1,4,6,7]. Monolayer VSe_2, especially in the light of recent works[12,18,19], seems to have a strong substrate-dependence. It is therefore likely that our calculation, which is based on freestanding VSe_2, does not capture the intricacies of all experimental systems. At the Fermi surface, an (additional) 4×1 CDW found on some substrates[1,6,12,19], might induce

an insulating state. A possible cause for the presence of different and competing CDW orders might be substrate-induced strain[20], which is not included in our DFT calculations. Supplementary Figure 13c shows the Fermi surface and constant-energy contours at higher binding energies of a VSe_2 system where the Fermi surface is fully gapped at $25\,\mathrm{K}$, taken from Ref. 7. In this case, though our calculation in Supplementary Figure 13d does not predict the fully gapped surface, we see that it captures the features of the band structure away from the Fermi level quite well. The dissimilarities between VSe_2 systems with different CDW orders might therefore pertain mostly to the Fermi surface and the unoccupied states.

References

1. Duvjir, G. *et al.* Emergence of a metal–insulator transition and high-temperature charge-density waves in VSe_2 at the monolayer limit. *Nano Lett.* **18**, 5432 (2018). URL https://doi.org/10.1021/acs.nanolett.8b01764.

2. Chen, P. *et al.* Unique gap structure and symmetry of the charge density wave in single-layer VSe_2. *Phys. Rev. Lett.* **121**, 196402 (2018). URL https://doi.org/10.1103/PhysRevLett.121.196402.

3. Coelho, P. M. *et al.* Charge density wave state suppresses ferromagnetic ordering in VSe_2 monolayers. *J. Phys. Chem. C* **123**, 14089 (2019). URL https://doi.org/10.1021/acs.jpcc.9b04281.

4. Wong, P. K. J. *et al.* Evidence of spin frustration in a vanadium diselenide monolayer magnet. *Adv. Mater.* **31**, 1901185 (2019). URL https://doi.org/10.1002/adma.201901185.

5. Bonilla, M. *et al.* Strong room-temperature ferromagnetism in VSe_2 monolayers on van der Waals substrates. *Nat. Nanotechnol.* **13**, 289 (2018). URL https://doi.org/10.1038/s41565-018-0063-9.

6. Chua, R. *et al.* Can reconstructed Se-deficient line defects in monolayer VSe_2 induce magnetism? *Adv. Mater.* **32**, 2000693 (2020). URL https://doi.org/10.1002/adma.202000693.

7. Feng, J. *et al.* Electronic structure and enhanced charge-density wave order of monolayer VSe_2. *Nano Lett.* **18**, 4493 (2018). URL https://doi.org/10.1021/acs.nanolett.8b01649.

8. Nomura, Y. & Arita, R. Ab initio downfolding for electron-phonon-coupled systems: Constrained density-functional perturbation theory. *Phys. Rev. B* **92**, 245108 (2015). URL https://doi.org/10.1103/PhysRevB.92.245108.

9. Berges, J., van Loon, E. G. C. P., Schobert, A., Rösner, M. & Wehling, T. O. Ab initio phonon self-energies and fluctuation diagnostics of phonon anomalies: Lattice instabilities from Dirac pseudospin physics in transition metal dichalcogenides. *Phys. Rev. B* **101**, 155107 (2020). URL https://doi.org/10.1103/PhysRevB.101.155107.

10. Giustino, F., Cohen, M. L. & Louie, S. G. Electron-phonon interaction using Wannier functions. *Phys. Rev. B* **76**, 165108 (2007). URL https://doi.org/10.1103/PhysRevB.76.165108.

11. Poncé, S., Margine, E., Verdi, C. & Giustino, F. EPW: Electron–phonon coupling, transport and superconducting properties using maximally localized Wannier functions. *Comput. Phys. Commun.* **209**, 116 (2016). URL https://doi.org/10.1016/j.cpc.2016.07.028.

12. Chua, R. *et al.* Coexisting charge-ordered states with distinct driving mechanisms in monolayer VSe_2 (2021). URL https://arxiv.org/abs/2104.12420.

13. Williams, P. M. Phase transitions and charge density waves in the layered transition metal dichalcogenides. In Lévy, F. (ed.) *Crystallography and Crystal Chemistry of Materials with Layered Structures* (Reidel, Dordrecht, 1976). URL https://doi.org/10.1007/978-94-010-1433-5_2.

14. Giuliani, G. & Vignale, G. *Quantum Theory of the Electron Liquid* (Cambridge University Press, Cambridge, 2005). URL https://doi.org/10.1017/CBO9780511619915.

15. Georges, A., Kotliar, G., Krauth, W. & Rozenberg, M. J. Dynamical mean-field theory of strongly correlated fermion systems and the limit of infinite dimensions. *Rev. Mod. Phys.* **68**, 13 (1996). URL https://doi.org/10.1103/RevModPhys.68.13.

16. Qazilbash, M. M. *et al.* Electronic correlations in the iron pnictides. *Nat. Phys.* **5**, 647 (2009). URL https://doi.org/10.1038/nphys1343.

17. Ugeda, M. M. *et al.* Characterization of collective ground states in single-layer $NbSe_2$. *Nat. Phys.* **12**, 92 (2016). URL https://doi.org/10.1038/nphys3527.

18. Zong, J. *et al.* Observation of multiple charge density wave phases in epitaxial monolayer 1T-VSe_2 film. *Research Square* (2021). URL https://doi.org/10.21203/rs.3.rs-498840/v1.

19. Duvjir, G. *et al.* Multiple charge density wave phases of monolayer VSe_2 manifested by graphene substrates. *Nanotechnology* **32**, 364002 (2021). URL https://doi.org/10.1088/1361-6528/ac06f3.

20. Si, J. G. *et al.* Origin of the multiple charge density wave order in 1T-VSe_2. *Phys. Rev. B* **101**, 235405 (2020). URL https://doi.org/10.1103/PhysRevB.101.235405.
