# Supplementary material for: A full gap above the Fermi level: the charge density wave of monolayer VS2
Source: Nat Commun. 2021 Nov 25;12:6837. doi: 10.1038/s41467-021-27094-x (PMC8617271; doi:10.1038/s41467-021-27094-x)
Supplement: Supplementary file 3 — Description of Additional Supplementary Files [file 41467_2021_27094_MOESM3_ESM.pdf]

Description of Additional Supplementary Files

File Name: Supplementary Movie 1:

Description: **a** Fermi surface, **b** band structure and density of states, **c** top view, and **d** side view of monolayer 1T VS_2 for atomic displacements toward the relaxed charge density wave structure. Calculations using density-functional theory have been performed for 0 %, 1/3, 2/3, and 100 % of the final displacements. In between, the Hamiltonian in the Wannier basis has been interpolated linearly.

File Name: Supplementary Movie 2:

Description: **a** Fermi surface, **b** band structure and density of states, **c** top view, and **d** side view of monolayer 1T-VS_2 for the projection of atomic displacements onto unstable phonon modes. Calculations using density-functional theory have been performed for 0 %, 1/3, 2/3, and 100 % of the final displacements. In between, the Hamiltonian in the Wannier basis has been interpolated linearly.

File Name: Supplementary Movie 3:

Description: **a** Fermi surface, **b** band structure and density of states, **c** top view, and **d** side view of monolayer 1T-VS_2 for the orthogonal complement of the atomic displacements. Calculations using density-functional theory have been performed for 0 %, 1/3, 2/3, and 100 % of the final displacements. In between, the Hamiltonian in the Wannier basis has been interpolated linearly.
